# Effect of dietary chitosan oligosaccharide supplementation on the pig ovary transcriptome

**DOI:** 10.1039/c7ra10172d

**Published:** 2018-04-10

**Authors:** Qingsong Xu, Chen Qu, Jin Wan, Gong Cheng, Wen Yang, Changhao Gong, Jun He, Yuguang Du

**Affiliations:** Liaoning Key Laboratory of Marine Animal Immunology and Disease Control, Dalian Ocean University 52. Heishijiao Street, Shahekou District Dalian 116023 China qingsongxu2003@163.com +86 411 84763004 +86 411 84763004; Institute of Animal Nutrition, Sichuan Agricultural University Chengdu 611130 China hejun8067@163.com +86-28-86290920 +86-13419354223; Institute of Process Engineering, Chinese Academy of Sciences Beijing 100190 China; Zhongke Runxin (Suzhou) Biological Technology Co., Ltd. Suzhou 215000 China

## Abstract

Fecundity improvement is one of the most important economic traits for the swine industry as it significantly increases production efficiency. Intriguingly, chitosan oligosaccharide (COS), a biomaterial with an active amino group, could promote sow reproductive performance. Therefore, we investigated the effects of dietary COS supplementation on the gene expression differences in the ovaries of sows using the RNA-Seq method. This analysis obtained 13 960 051 and 14 564 863 clean reads in control ovary and COS ovary libraries, respectively. A total of 486 differentially expressed genes (DEGs) were thereby identified (FDR ≤ 0.001, |log_2_ ratio| ≥ 1). There were 234 up-regulated and 252 down-regulated genes in the COS ovary samples compared with the control ovary samples. A large number of these DEGs were involved in the terms cellular process, cell & cell part and binding. Furthermore, pathway analysis indicated that these DEGs were significantly enriched in 34 Kyoto Encyclopedia of Genes and Genomes (KEGG) pathways, including cell cycle, progesterone-mediated oocyte maturation, metabolic pathways, oocyte meiosis, and hematopoietic cell lineage among others. These results provided the molecular mechanisms of using COS feed additive for improving sow litter size and prolificacy.

## Abstract 2

Fecundity improvement is one of the most important economic traits for the swine industry. In this study, we identified 486 differentially expressed genes associated with sow prolificacy from COS administrated sow ovaries by RNA-seq.

## Graphical abstract

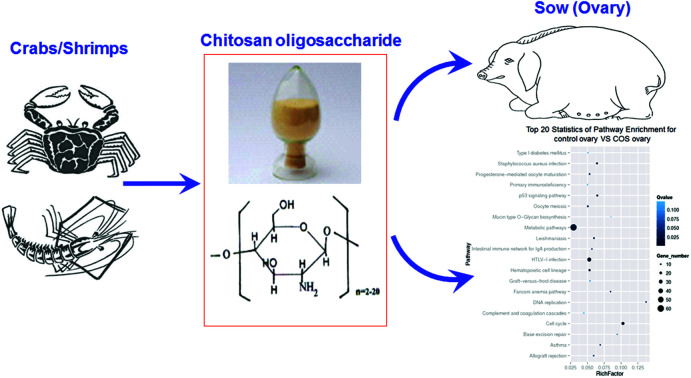

## Introduction

In the swine production industry, litter size is one of the most meaningful economic traits and varies among individual animals. However, reproductive traits in swine are complex, from ovulation, fertilization, and implantation through to the birth of piglets, every process may affect litter size.^1^ Recently, various efforts have been made to investigate factors influencing litter size, including genetic factors, management of sows, optimizing nutrition and husbandry.^2^ It is well known that sow diet and health during gestation are important for foetal survival and sow reproductive performance.^3^ Nutrition optimization for increased litter size has in turn improved the prolificacy of sows over the past ten years. For instance, some functional oligosaccharides^4^ and amino acid^5^ have been used to improve the pregnant animal reproductive performance. Nonetheless, the molecular basis of nutrition improvement and sow prolificacy remains largely unknown.

With the rapid development of sequencing technique and bioinformatics analysis, RNA-Seq technology provides a platform for measuring large-scale gene expression pattern.^6^ It has many advantages such as more accurate quantization, a wider testing range, higher repeatability, and more reliable analysis.^7^ Currently, in order to investigate the novel transcript units and differentially expressed genes (DEGs), the RNA-Seq has been widely applied to domestic animals, such as pig,^8^ cow,^9^ goat,^10^ sheep,^11^ and others. In addition, the efficacy of RNA-Seq has also been used in mammalian reproductive tissues, including pig ovary,^12^ pig placenta,^13^ bovine blastocyst,^14^ goat ovary^10^ and sheep ovary.^11^ Therefore, using RNA-Seq technology to study the relationship between nutrition improvement and sow prolificacy trait is the Frontier research of animal nutrition.

Chitosan oligosaccharide (COS), which is a depolymerized product of chitosan, possesses significant applications in the pharmaceutical, food, agricultural and environmental industries.^15,16^ Particularly, COS has potential applications as a dietary supplement or nutraceutical for animals.^17^ Several studies have demonstrated that dietary supplementation with COS could improve piglets growth performance and intestinal development^18^ while enhancing animal health.^19,20^ Our previous studies also indicated that dietary COS supplementation improved foetal survival and reproductive performance in multiparous sows,^21,22^ and the foetal survival rate in sows after 35 days COS supplementation was elevated by approximately 13.0%.^22^ Appreciation of the important role of COS in regulating mammalian foetal survival and growth rates has grown steadily in recent years. Maternal COS supplementation provides an important breakthrough for developing strategies to reduce prenatal loss. Nevertheless, understanding the variation in the expression of genes responsible for COS-induced foetal survival and growth alterations is in its primitive stage. In this study, we detected the differential expression profiling of the mRNAs in two groups (control sow ovary and COS sow ovary) using RNA-Seq technology. This work advanced our understanding of the molecular mechanism of COS-induced fecundity, and provided basic data for future studies.

## Materials and methods

### Preparation and composition of COS

COS was prepared by the enzymatic hydrolysis of chitosan according to the method we reported previously.^23^ The products of the enzymolysis were a mixture of several oligosaccharides with a degree of deacetylation over 95% and an average molecular weight ≤ 1000 Da. The percentage composition of COS was 3.7%, 16.1%, 28.8%, 37.2% and 14.2%, with a degree of polymerization (DP) of 2–6.

### Animals treatment and ovary collection

Twenty-four multiparous sows (Yorkshire; high-prolificacy gilts introduced to China from Canada), whose parities were in the range 3–4, were selected from a commercial pig farm (Leshan, China) and transported to Sichuan Agricultural University (Chengdu, China). The sows were individually housed in gestation crates (1.5 × 2.0 m) in a pregnancy room. The ambient temperature in the pregnancy room was maintained at 15–18 °C. All sows were determined to be in the oestrous stage and then were inseminated twice with unfrozen semen *via* artificial insemination 3–5 days after weaning. The sows were randomly allotted to one of two treatments (12 sows/treatment) from day 1 of mating to ensure that each group had the same number of sows of similar parity. The treatment groups were as follows: (1) control diet without supplementation (CON); (2) control diet with COS added at a concentration of 100 mg kg^−1^ (COS). The sows were fed twice daily either 2.2 kg of control or COS-supplemented diets during days 1 to 34 of gestation (at 08 : 00 and 18 : 00). In addition, all sows were given *ad libitum* access to water.

At day 35 of gestation, 12 hours after their last meal, six sows were euthanized with an intravenous injection of pentobarbital sodium (50 mg kg^−1^ body weight) for 15 min and then the abdomens were immediately opened. Their intact ovaries were rapidly harvested from their carcasses and immediately frozen in liquid nitrogen. All tissue samples were stored at −80 °C until the total RNA extraction procedure was performed.

### Library preparation and sequencing

The ovaries were completely ground and total RNA was extracted using TRIzol (Invitrogen, Carlsbad, CA, USA). The quality of the total RNA (RNA Integrity Number ≥ 7) was checked using the Agilent 2100 Bioanalyzer system (Santa Clara, CA, USA). Total RNA from three ovary samples was pooled prior to library preparation in the two experimental groups. Equimolar quantities of RNA from each ovary sample were combined into one pool. According to the manufacturer's manual, sequencing libraries were performed at Beijing Genomics Institute (BGI, Shenzhen, China) using the Illumina Truseq RNA Sample Preparation Kit (Illumina, San Diego, USA). Briefly, mRNA was first extracted from total RNA using oligo (dT) magnetic beads and sheared into short fragments of about 200 bases. These fragmented mRNAs were then used as templates for cDNA synthesis. The cDNAs were then PCR amplified to complete the library. The cDNA libraries were sequenced using an Illumina HiSeq™ 2000 platform.

### Bioinformatics analysis

All clean reads were obtained by rejecting low quality sequence or sequencing adapters, and reads with more than 10% unknown nucleotides (N). The clean reads were aligned to the gene sequences (downloaded from NCBI database) and pig genome (*Sus scrofa* 10.2) through TopHat software,^24^ allowing up to two base mismatches. Unmapped or multi-position matched reads were excluded from further analyses. Moreover, sequence saturation analyses of the two libraries were executed to provide an overview of the project.

### Expression profiling

The number of mapped reads for each gene was normalized and calculated by using the reads per kilo bases per million reads (RPKM) method, which is an effective method for eliminating the influence of sequencing discrepancy and gene length.^25^ The false discovery rate (FDR) ≤ 0.001 and |log_2_ ratio| ≥ 1 were used to identify differentially expressed genes (DEGs).^26^

### Gene ontology and pathway enrichment analysis of differentially expressed genes

All DEGs were submitted to the databases of Gene Ontology (GO) and Kyoto Encyclopedia of Genes and Genomes (KEGG) for enrichment analysis. GO analysis was performed using the Blast 2 GO software^27^ to annotate the function of these DEGs. In addition, the KEGG database was used for the DEGs enriched pathway analysis (http://www.genome.ad.jp/kegg/). Pathways with a *Q* value ≤ 0.05 was defined as a significantly enriched pathway in terms of DEGs.

### Validation of RNA-Seq data

To validate the DEGs identified by RNA-Seq, nine genes (Table 2) revealed to be differentially expressed were chosen randomly for qPCR validation. Ovarian total RNA isolation, cDNA synthesis and qPCR analysis were conducted as previously described.^28^ The primers used for qPCR analysis were listed in Table 2 with β-actin identified as a reference control. The qPCR reactions were performed on an ABI StepOnePlus™ Real-Time PCR System (ABI, USA) using a SYBR Green qPCR Mix (Takara, Dalian, China) in accordance with the manufacturer's instructions. The thermal cycling conditions were 95 °C for 10 min, followed by 40 cycles of 95 °C for 15 s and 60 °C for 1 min. Relative quantification analyses were used the comparative CT method, and relative gene expression levels were calculated using the 2^−ΔΔ*C*_T_^ method.^29^

## Results

### Overview of sequencing data

**Fig. 1:**
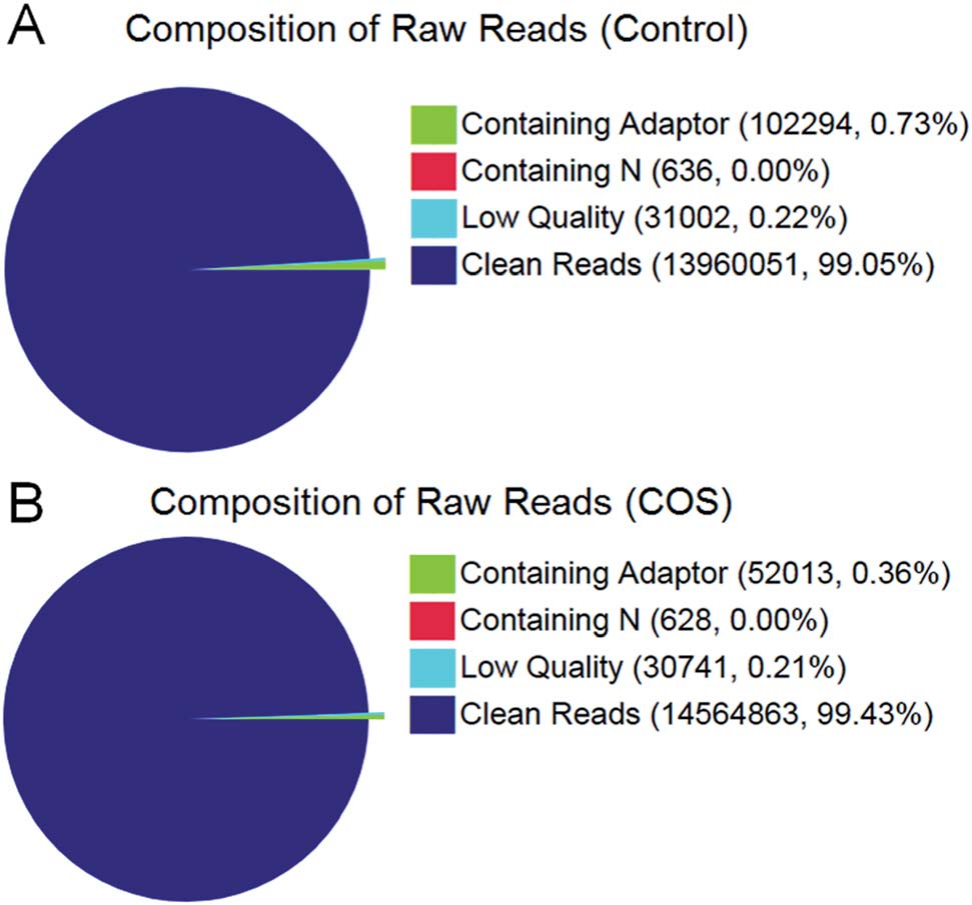
Composition of total raw reads from the control sow ovary (A) and COS sow ovary (B) libraries.

**Fig. 2:**
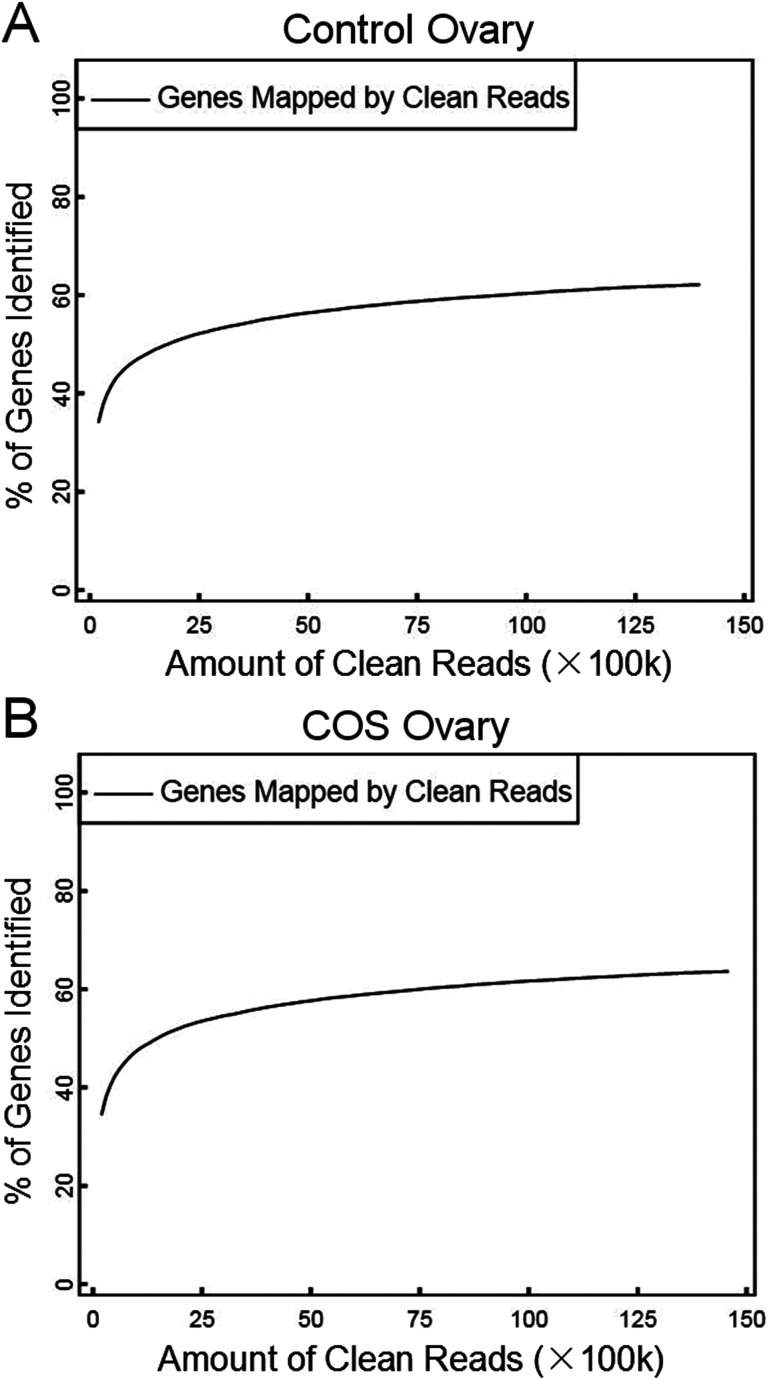
Saturation description of control sow ovary (A) and COS sow ovary (B). The number of detected genes continued increasing as the total number of sequencing reads increased. When the number of reads reached a certain amount, the number of detected genes almost ceased increasing.

In the present study, we constructed two RNA-Seq libraries from the control sow ovary and COS sow ovary, respectively. After filtering the adaptor sequences, low quality sequences and regions containing N sequences, the two libraries still generated over 1.39 Gb clean reads in each library, and the percentages of clean reads among the raw reads reached 99.05% and 99.43% in the control ovary and COS ovary libraries, respectively (Fig. 1), which demonstrated that the two libraries were of high-quality. The major characteristics of the two libraries were analyzed in Table 1. The result showed varying amount of sequencing reads for these samples. In both libraries, although 59.30% of the reads in control sow and 56.92% of the reads in COS sow could be mapped to reference genes, approximately 74% of the reads mapped to pig genome (control sow ovary with 73.50%, COS sow ovary with 74.57%). For the unique match, a little more than 45% and 66% of the reads corresponded to reference genes and genome respectively. Moreover, 48.45% of the reads in control sow and 46.70% of the reads in COS sow could be perfectly matched to the reference genes, and approximately 58% of the reads perfectly matched to genome. In addition, the results of saturation analyses (Fig. 2) demonstrated that when the number of sequenced reads reached 2.5 M or more, the number of detected genes almost ceased increasing, which validated the integrity of the libraries for use in further analysis.

**Table 1:** A summary of the sequencing reads alignment to the *Sus scrofa* genome and reference genes

Sample	Alignment to genome		Alignment to reference genes	
	Control	COS	Control	COS
Total reads	13 960 051	14 564 863	13 960 051	14 564 863
Total base pairs	684 042 499	713 678 287	684 042 499	713 678 287
Total mapped reads	10 261 061(73.50%)	10 860 921(74.57%)	8 278 882(59.30%)	8 289 979(56.92%)
Perfect match	8 048 824(57.66%)	8 581 743(58.92%)	6 763 308(48.45%)	6 802 338(46.70%)
≤2bp Mismatch	2 212 237(15.85%)	2 279 178(15.65%)	1 515 574(10.86%)	1 487 641(10.21%)
Unique match	9 332 910(66.85%)	9 875 898(67.81%)	6 605 864(47.32%)	6 605 721(45.35%)
Multi-position match	928 151(6.65%)	985 023(6.76%)	1 673 018(11.98%)	1 684 258(11.56%)
Total unmapped reads	3 698 990(26.50%)	3 703 942(25.43%)	5 681 169(40.70%)	6 274 884(43.08%)

### Identification and analysis of DEGs

**Fig. 3:**
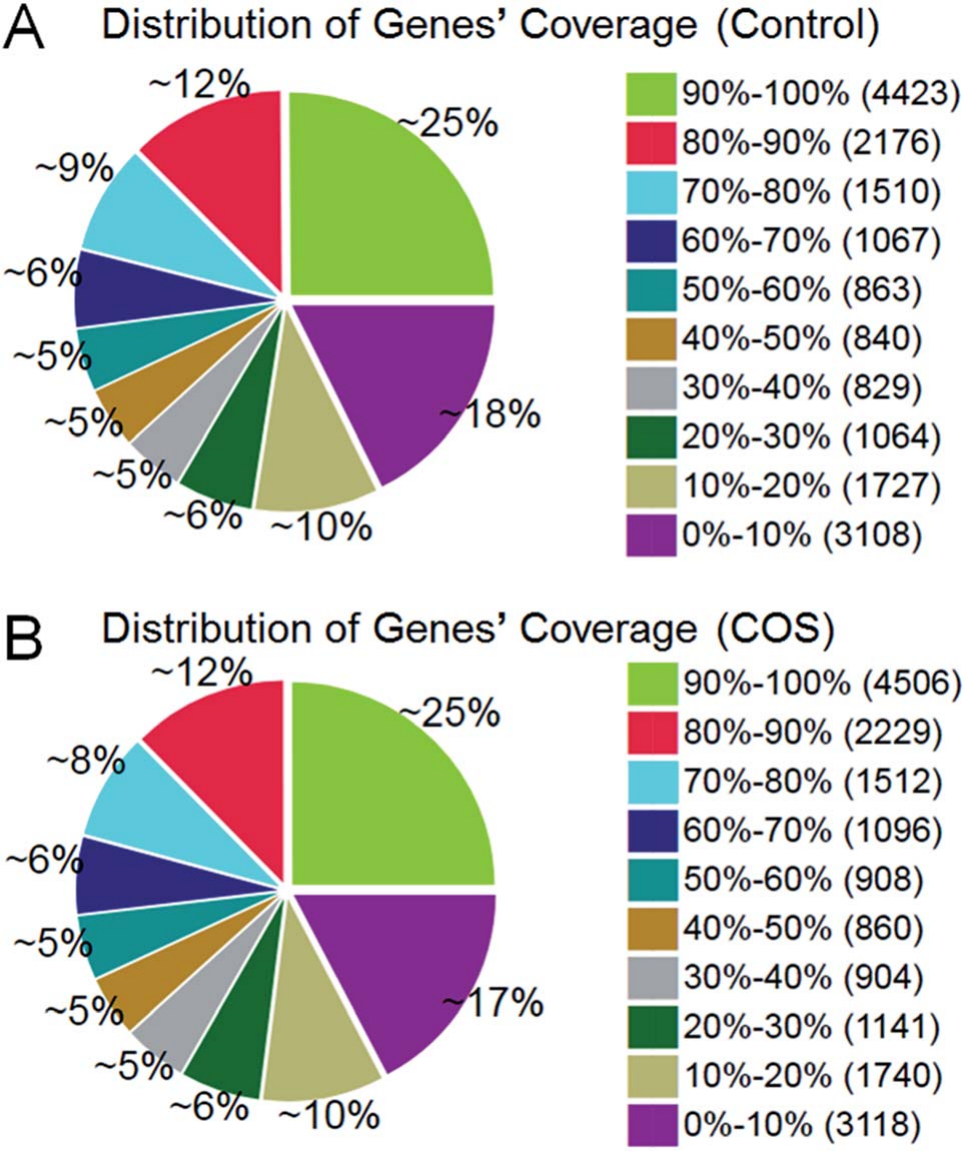
Distribution of genes coverage in the two sow ovary libraries. Gene coverage was the percentage of a gene covered by reads. This value was equal to the ratio of total base count in a gene covered by uniquely mapped reads to the total base count for that gene.

**Fig. 4:**
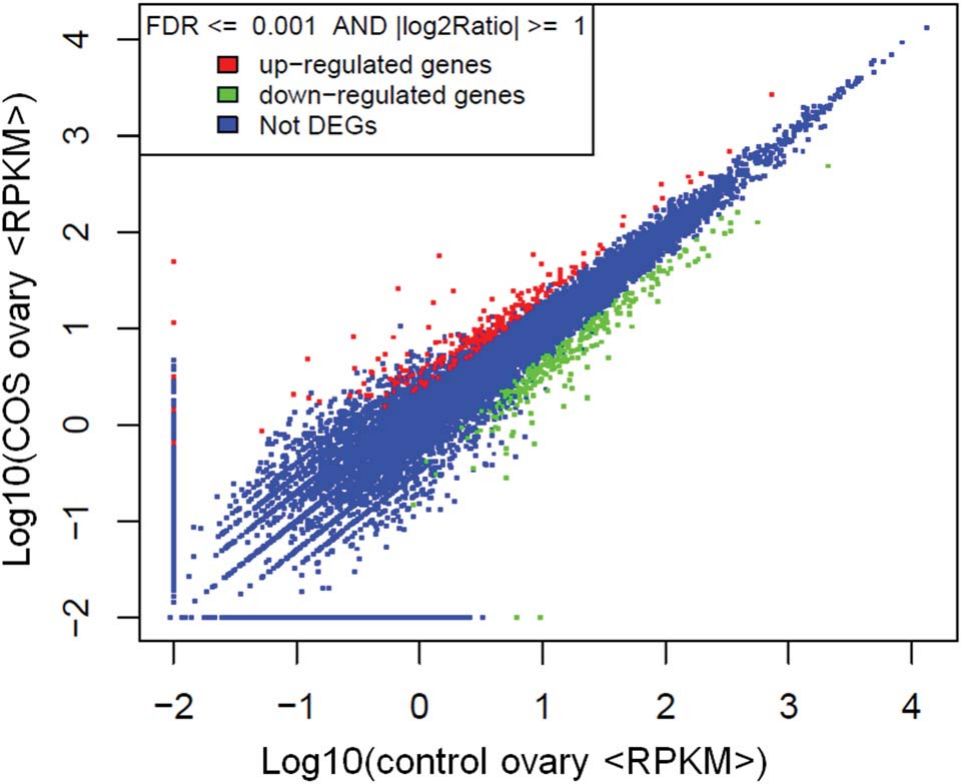
Scatter plot indicated the comparative results of log transformed gene expression levels and differentially expressed gene distributions between the two libraries.

The RPKM method was adopted to evaluate the gene expression levels. As a result, 17 607 and 18 014 reference genes were identified from control sow ovary and COS sow ovary libraries, respectively, which shared 16 741 genes in common. As shown in Fig. 3, 25% of the reference genes had 90–100% coverage, and 12% of the genes had 80–90% coverage in control ovary and COS ovary libraries, suggesting that the read distributions were similar between the two libraries. To identify the significance of differences in expressed genes, FDR ≤ 0.001 and the absolute value of log_2_ ratio ≥ 1 were defined as the threshold. A total of 486 significantly differentially expressed genes were identified between the two libraries, with 234 genes up-regulated and 252 genes down-regulated in COS ovary compared with control ovary (Fig. 4).

### Gene ontology and pathway enrichment analysis

**Fig. 5:**
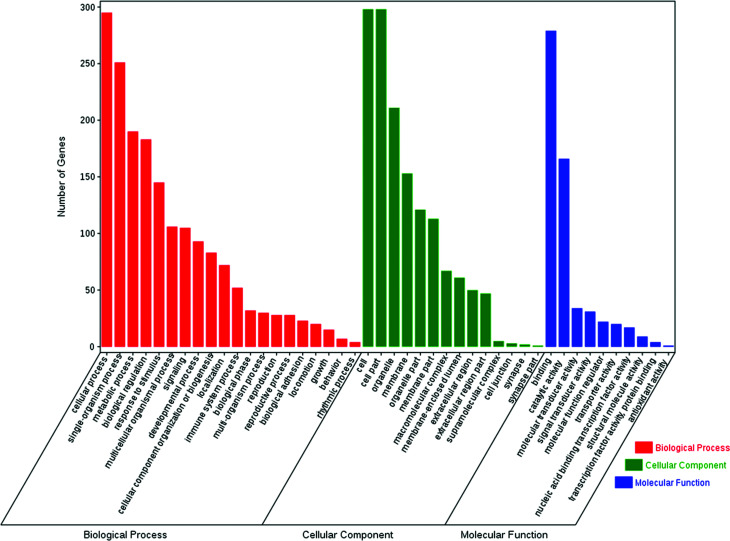
GO analysis of DEGs between the control and COS ovary libraries. The DEGs were classified into three categories: cellular component, molecular function, and biological process. The number of genes in each category were shown above.

The enrichment of DEGs in GO terms was analyzed to obtain insights into the biological implications. Several GO terms significantly enriched for DEGs were investigated and shown in Fig. 5. The GO annotation demonstrated that the DEGs were involved in many biological processes, such as cellular process, single-organism process, metabolic process, biological regulation, response to stimulus, multicellular organismal process, signaling, and developmental process. The main functional groups of DEGs in cellular component were cell, cell part, organelle, membrane, and organelle part, and in molecular function were binding and catalytic activity.

**Fig. 6:**
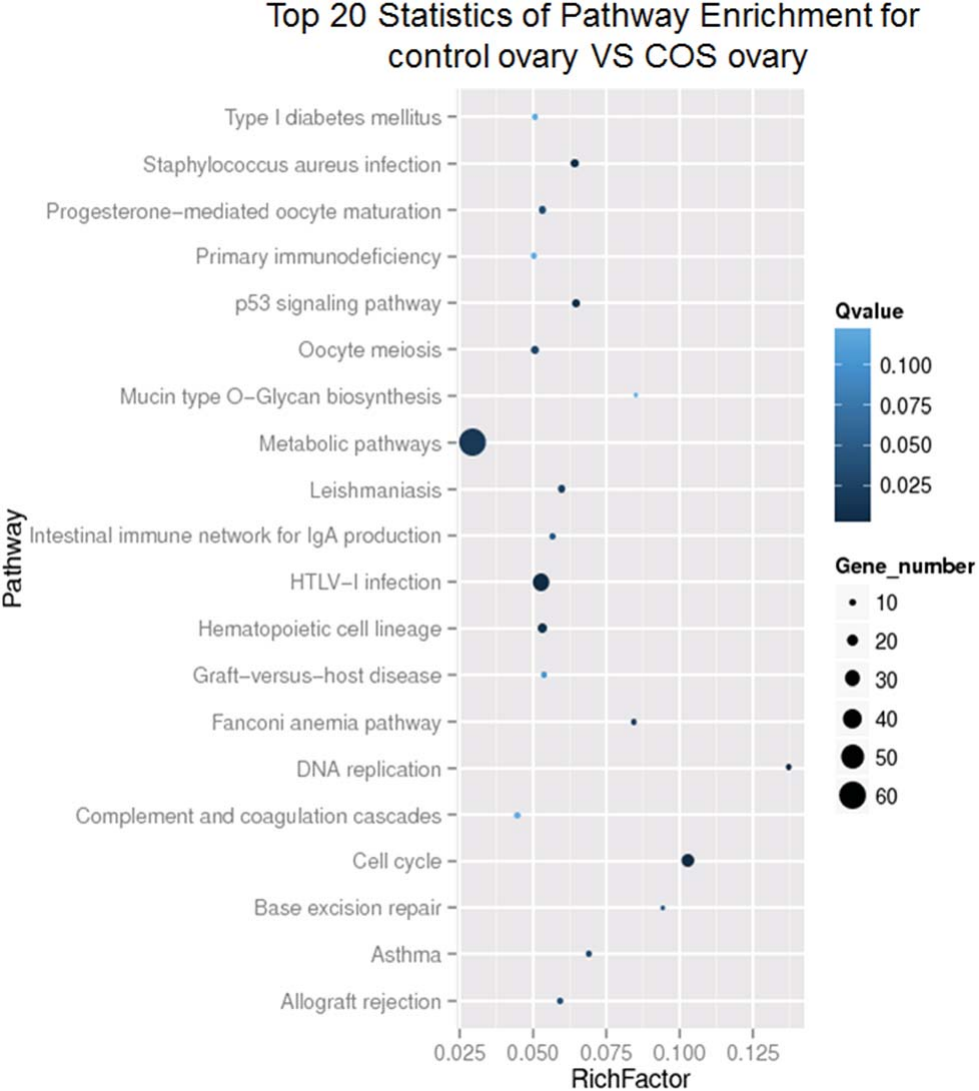
KEGG enrichment pathway analysis from DEGs. The ordinate represented the enriched pathway terms, and the abscissa represented the richness factor of these terms. Spot size represented the number of differentially expressed genes enriched in each pathway, while the color shade of the spot represented the *Q* value of each pathway (its less value means greater intensiveness).

According to the KEGG pathway database, the pathway analysis was performed to predict the significantly enriched signal transduction pathways and metabolic pathways in DEGs. After pathway enrichment analysis, 409 DEGs had been annotated in KEGG pathway. The results indicated that the significant signaling pathways were 34 pathways. For example cell cycle, progesterone-mediated oocyte maturation, p53 signaling pathway, DNA replication, metabolic pathways, oocyte meiosis, and hematopoietic cell lineage were among the most enriched pathways (Fig. 6).

### Confirmation of DEGs by real-time quantitative PCR

Nine DEGs were randomly selected for qPCR analysis to validate the expression patterns obtained by RNA-Seq. The results indicated that relaxin 2 (RLN2), lysozyme (LYZ), wnt family member 2 (WNT2), integrin subunit beta like 1 (ITGBL1), and endothelin receptor type B (EDNRB) were up-regulated and surfactant protein C (SFTPC), matrix metallopeptidase 9 (MMP9), E2F transcription factor 1 (E2F1), and cyclin B1 (CCNB1) were down-regulated in COS ovary samples (Table 2), which were basically consistent with the RNA-Seq results. Therefore, RNA-Seq can be used to reliably and accurately perform for mRNA differential expression analysis.

**Table 2:** Validation of selected RNA-Seq genes expression by real-time RT-PCR analysis

Gene ID	Description	log_2_ Ratio (COS ovary/Control ovary)		Regulation	Primer sequence
		RNA-Seq	qPCR		
ENSSSCT00000005749	Relaxin 2 (RLN2)	5.30	4.02	Up	F:CTGAAGGCAACATTGTCTGA
					R:TCTCTTTTTTCTGGAATGTTTAT
ENSSSCT00000000530	Lysozyme (LYZ)	3.85	3.13	Up	F:GCCAAGTGGGAAAGTGA
					R:AGGTCATCGTCCAGCAA
ENSSSCT00000018104	Wnt family member 2 (WNT2)	2.09	1.50	Up	F:TGTGACCCGAAGAAGAAGG
					R:ACCGCTTTACAGCCTTCC
ENSSSCT00000010444	Integrin subunit beta like 1 (ITGBL1)	1.83	1.11	Up	F:AGACCTACGACGGCAGCAC
					R:TACTTTTTTTCTTGGTCAGGTCAC
ENSSSCT00000010390	Endothelin receptor type B (EDNRB)	1.37	1.42	Up	F:TCCGTGCGAAGGACCCA
					R:ATGTGAAGCAGGTCTCCCAG
ENSSSCT00000010544	Surfactant protein C (SFTPC)	−3.60	−2.23	Down	F:AGAAACATACTGAGATGGTCCTA
					R:AGCCGCTGGTAGTCATAGA
ENSSSCT00000008139	Matrix metallopeptidase 9 (MMP9)	−2.52	−2.29	Down	F:AGCCCTGCGTGTTTCCA
					R:CGAGTTGCCTCCCGTCA
ENSSSCT00000007953	E2F transcription factor 1 (E2F1)	−1.98	−1.86	Down	F:CTGACCACCAAACGCTTCC
					R:TGCCTAGCCACTGGATGTG
ENSSSCT00000024108	Cyclin B1 (CCNB1)	−1.82	−1.49	Down	F:CAAATCAGGCAGATGGAAAT
					R:TCTGAGAAGGAGGAAAGTGC
	β-Actin				F:CGAGCGCTTCCGGTGTCCAG
					R:GTGGTCCCGCCAGACAGCAC

## Discussion

As the safe and burgeoning feed additive, COS has attracted more and more attentions recently, because it is not only easily soluble in water (generally, the MW of COS is 10 kDa or less) and free amino groups in d-glucosamine units, but also readily absorbed through the intestine, quickly getting into the blood flow.^30^ Most importantly, COS is renewable, non-toxic, biocompatible, and biodegradable.^15^ Our previous study demonstrated that COS supplementation increased the total number of piglets born by 18.5%, the number of piglets born alive by 19.2%, and the live born litter weight by 31.3%.^21^ Moreover, we found the foetal survival rate in pregnant sows after 35 days COS supplementation was elevated by approximately 13.0%.^22^ However, the underlying molecular mechanism of COS feed additive in pregnant sow remains largely unknown, especially the relative genes variation. As the ovary directly mediates ovulation and affects litter size, it has a significant impact on the fecundity of mammals.^31^ In the present study, we identified 486 differentially expressed genes in ovaries in control sow and COS sow groups using RNA-Seq technology. A large number of these DEGs were involved in the terms cellular process, cell & cell part and binding. Furthermore, pathway analysis indicated that these DEGs were significantly enriched in cell cycle, progesterone-mediated oocyte maturation, metabolic pathways, oocyte meiosis, or hematopoietic cell lineage and so on.

Previous studies of pig ovaries indicated that the most differentially expressed genes identified by RNA-Seq were likely to be significant for improving litter size.^12^ In this study, 486 DGEs in ovaries of control sow and COS sow groups were identified by RNA-Seq. Some of the DGEs corresponding to genes previously were involved in prolificacy processes, such as relaxin 2,^32^ placenta specific 8 (PLAC8),^33^ wnt family member 2 (WNT2)^34^ and vascular endothelial growth factor (VEGF)^35^ were up-regulated in COS sow ovary. In animals, relaxin softens the cervix (cervical ripening), and relaxes the uterine musculature. Thus, for a long time, relaxin has been regarded as a pregnancy hormone.^32^ PLAC8 has been investigated in embryo development in different species, and its distribution in cells is dynamic and highly regulated in a manner depending on the developmental stage and cell type.^33^ The Wnt gene family consists of structurally related genes that encode secreted signaling proteins involved in the Wnt signaling pathway. These proteins have been associated with several developmental processes, including regulation of cell fate and patterning during embryogenesis.^34^ It has been demonstrated that VEGF system constitute the most important signaling pathway in angiogenesis, and play an important role in the female ovulatory cycle including follicular development, ovulation, and corpora lutea formation.^35^ Furthermore, it is now increasingly clear that the feto-placental unit survival and growth are influenced by a complex interactive network of cytokines, some of which are produced by local immune components and others by reproductive tissues.^36^ In contrast to control sow, there was a particularly high overrepresentation of genes related to the immune response in COS sow, such as CD19, CD48, CD84, CD3E, C3a receptor, C5, and lysozyme.

To investigate the biological functions of the DEGs, we performed the GO annotation and KEGG pathway analysis. The results demonstrated that some DEGs between control and COS sows were mainly in the cell cycle, progesterone-mediated oocyte maturation, oocyte meiosis, hematopoietic cell lineage, or metabolic pathways, and so on. Oocyte maturation and early embryo development require precise coordination between cell cycle progression and the developmental programme. The tempo of oocyte meiotic and embryonic mitotic divisions is set by the rate of cyclin B accumulation and the timing of its destruction.^37^ Progesterone production from the corpus luteum is critical for oocyte maturation and natural reproduction. Luteal phase deficiency in natural cycles is an important cause of infertility and pregnancy loss.^38^ It is well established that pubertal activation of the reproductive axis and maintenance of fertility are critically dependent on the magnitude of body energy reserves and the metabolic state of the organism.^39^ As paradigmatic example, much has been learned on the reproductive roles of key metabolic hormones (such as leptin, insulin and ghrelin).^40^ The DEGs were not only related to reproduction-related pathways but also in those associated with nutrient metabolism. This indicated that the genes might be involved in both reproduction and metabolism. The molecular regulation of animal traits is very complex and the relationships between genes and traits are usually that of “many-to-one” or “one-to-many”.^12^ It was readily appreciated that, most physiological processes interacted with each other in the life of organisms.

## Conclusion

In summary, we identified 486 DEGs associated with litter size from COS administrated pig ovaries, and a large number of these DEGs were involved in cell cycle, progesterone-mediated oocyte maturation, metabolic pathways, oocyte meiosis, or hematopoietic cell lineage and so on. These novel findings not only furthered our understanding of the molecular mechanisms underlying the beneficial effects of COS on foetal survival and reproductive performance in pregnant sows, but also provided a theoretical basis for developing functional carbohydrates such as COS as green feed additives for the livestock industry.

## Conflicts of interest

The authors declare that they have no competing interests.

## Ethical statement

All animal procedures and sample collection were conducted according to the Regulations for the Administration of Affairs Concerning Experimental Animals (approved by the State Council of the People's Republic of China on October 31, 1988 and promulgated by Decree No. 2 of the State Science and Technology Commission on November 14, 1988, and revised on March 1st, 2017), and was also approved by the Animal Care and Use Committee of Sichuan Agricultural University (Sichuan, China).

## Supplementary Material
